# Microbiota-driven therapeutic efficacy of Hyperoside in ulcerative colitis and associated anxiety

**DOI:** 10.3389/fcimb.2026.1734356

**Published:** 2026-01-29

**Authors:** Li Yin, Lin Xu, Yu-nan Shan, Zhilin He, Yanbin Li, Wei Chen

**Affiliations:** 1 Department of Gastroenterology, Shanghai Sixth People’s Hospital Affiliated to Shanghai Jiao Tong University School of Medicine, Shanghai, China; 2 The First Clinical Medical College, Shandong University of Traditional Chinese Medicine, Jinan, Shandong, China; 3 School of Traditional Chinese Medicine, Hubei University of Chinese Medicine, Wuhan, Hubei, China; 4 Department of Neurology, The First Affiliated Hospital of Shandong First Medical University & Shandong Provincial Qianfoshan Hospital, Shandong Institute of Neuroimmunology, Shandong Key Laboratory of Rheumatic Disease and Translational Medicine, Jinan, Shandong, China

**Keywords:** anxiety, arginine biosynthesis, gut microbiota, gut-brain axis, hyperoside, MAPK/PI3K-Akt/NF-κB signaling pathways, ulcerative colitis

## Abstract

**Background:**

Ulcerative colitis (UC) is subtype of inflammatory bowel disease that is frequently comorbid with anxiety disorders. However, effective dual-targeting therapies are still lacking. Hyperoside (HYP), a natural flavonoid, exhibits anti-inflammatory and neuroprotective properties, yet its potential therapeutic effects on UC and associated anxiety, as well as the underlying mechanisms, remain largely unexplored.

**Methods:**

A murine model of DSS-induced colitis was established and treated with HYP. Disease activity was assessed through body weight, colon length, and histopathology. Anxiety-like behaviors were evaluated using open field and elevated plus maze tests. Neuroinflammation was examined through immunohistochemistry of BDNF expression and microglial activation. Gut microbiota composition was profiled by metagenomic sequencing, and metabolomic profiling was conducted using the Q300 Kit. Network pharmacology and molecular docking were employed to predict signaling pathways, which were further validated by Western blotting. Additionally, antibiotic depletion experiments were conducted to determine microbiota dependency.

**Results:**

HYP administration significantly ameliorated DSS-induced colitis, as evidenced by attenuated weight loss, restored colon length, and improved histopathology. It suppressed pro-inflammatory cytokines (TNF-α, IL-6, IL-1β) and restored intestinal barrier integrity by upregulating Mucin-2 and ZO-1. Furthermore, HYP also alleviated anxiety-like behaviors and mitigated neuroinflammation by increasing BDNF levels and suppressing microglial activation. HYP treatment also restored gut microbial homeostasis, enriching beneficial bacteria such as *Enterobacter ludwigii* while reducing the abundance of *Enterobacter hormaechei, Escherichia coli*, and *Acinetobacter baumannii*. Metabolomic analysis revealed that HYP significantly promoted arginine biosynthesis. Network pharmacology and molecular docking identified the MAPK, PI3K-Akt, and NF-κB pathways as potential targets, with HYP showing strong binding affinity to MAPK3, AKT1, and NFκB1. Importantly, the therapeutic effects of HYP were abolished in microbiota-depleted mice.

**Conclusion:**

Our findings demonstrate that HYP effectively alleviates DSS-induced colitis and comorbid anxiety-like behaviors. Its efficacy is dependent on the gut microbiota and is associated with the restoration of microbial homeostasis, enhancement of arginine metabolism, and modulation of the MAPK/PI3K-Akt/NF-κB signaling pathways. HYP represents a promising microbiota-targeting therapeutic candidate for UC and its neuropsychiatric comorbidities.

## Introduction

Ulcerative colitis (UC), a major subtype of inflammatory bowel disease (IBD), is a chronic, idiopathic inflammatory disorder affecting the colonic mucosa, typically manifesting as abdominal pain, chronic diarrhea, rectal bleeding, and weight loss (Wangchuk et al., 2024). Over recent decades, the global prevalence of UC has increased markedly, imposing a growing clinical and economic burden (Wangchuk et al., 2024). Notably, UC is frequently complicated by psychiatric comorbidities, with studies reporting anxiety symptoms in 34.2% of patients and depressive symptoms in 24.0% (Barberio et al., 2021), highlighting the critical role of the gut-brain axis in disease progression.

Although the precise pathogenesis of UC remains incompletely understood, emerging evidence implicates multifactorial mechanisms, including intestinal barrier dysfunction, immune dysregulation, gut microbiota imbalance, and altered microbial metabolites (Liang et al., 2024). Of particular interest is the gut-brain axis, whose dysfunction may contribute to neuropsychiatric manifestations in UC patients. Disruption of intestinal barrier integrity increases permeability, facilitating the translocation of microbial components into systemic circulation. These microbial products can subsequently cross the blood-brain barrier, triggering neuroinflammatory responses that contribute to emotional and behavioral disturbances (Gracie et al., 2018).

The gut microbiota communicates with the central nervous system through multiple pathways, such as microglial activation and modulation of neurotransmitter systems, including serotonin, dopamine, and gamma-aminobutyric acid (GABA) (Sharon et al., 2016). This bidirectional interaction creates a vicious cycle: UC-associated dysbiosis exacerbates neuropsychiatric symptoms, while psychological stress and mood disorders in turn aggravate intestinal inflammation and mucosal damage (Gracie et al., 2018; Fairbrass et al., 2022; Li et al., 2024). Current UC therapies, though effective for managing intestinal inflammation, often show limited efficacy against psychiatric comorbidities and may induce significant adverse effects (Horst et al., 2015). Therefore, there is an urgent need to develop novel therapeutic strategies that can simultaneously target gut inflammation and associated anxiety disorders by restoring gut microbiota homeostasis.

Hyperoside (HYP), a flavonol glycoside also known as quercetin-3-O-galactoside, is widely present in medicinal plants such as *hypeticum perforatum* (Galeotti, 2017), *Crataegus pinnatifida Bge*, *Forsythia suspensa*, and *Cuscuta chinensis Lam* (Wang et al., 2022). Accumulating evidence suggests that HYP exhibits diverse pharmacological properties, including anti-inflammatory, anticancer, antibacterial, antiviral, antidepressant, and organ protective effects (Wang et al., 2022). Notably, preclinical studies have demonstrated that HYP protects against dextran sulfate sodium (DSS)-induced colitis (Cheng et al., 2021) and alleviates depressive-like behavior in social defeat models by modulating microglial polarization and neuroinflammation (Zhao et al., 2025). These findings suggest that HYP holds promise as a dual-target therapeutic agent for inflammation and anxiety-related disorders.

Given the interplay between UC and neuropsychiatric symptoms, as well as the potential of HYP to modulate both inflammatory and behavioral responses, we hypothesized that HYP alleviates UC and anxiety-like behaviors by modulating the gut microbiota and its associated metabolites, thereby influencing critical inflammatory signaling pathways. In this study, we employed a DSS-induced murine model of colitis to investigate the effects of HYP on intestinal pathology, anxiety-like behaviors, neuroinflammation, gut microbiota composition, and host metabolism. We further integrated network pharmacology and molecular docking to predict and validate potential signaling pathways and conducted antibiotic depletion experiments to conclusively assess the role of the gut microbiota in mediating the therapeutic effects of HYP.

## Materials and methods

### Reagents

Hyperoside was purchased from MUST Biotechnology Co., Ltd. (Chengdu, China). DSS (MW 36,000~50,000, 160110, MP) was obtained from MP Biomedicals (CA, USA). The following antibodies were used in this study: anti-ZO-1 antibody (AF5145, Affinity), anti-mucin-2 antibody (GB11344, Serbicebio), anti-BDNF antibody (GB11559, Serbicebio), anti-GFAP antibody (GB11096, Serbicebio), anti-Iba-1 antibody (GB154490, Serbicebio), NF-κB p65 (Proteintech, 10745-1-AP), Phospho-NF-κB p65 (Affinity, AF2006), AKT (Proteintech, 10176-2-AP), Phospho-AKT (Proteintech, 28731-1-AP), p38MAPK (Proteintech, 51115-1-AP), Phospho-p38MAPK (Affinity, AF4001). Antibiotics for microbiota depletion, including ampicillin (A102048), metronidazole (B300250), vancomycin (V301569),and neomycin (N412785) were purchased from Shanghai Aladdin Biochemical Technology Co., Ltd (Shanghai, China).

### Animals and treatments.

Male C57BL/6 mice (6–8 weeks old) were obtained from Shanghai SLAC Laboratory Animal Co. Ltd. and maintained under specific pathogen-free (SPF) conditions with a controlled environment (12 h light/dark cycle, 25 ± 2°C, 50–55% humidity). All experimental procedures were approved by the Ethical Review Committee of The First Hospital Affiliated with Shandong First Medical University & Shandong provincial Qianfoshan Hospital (application number: 2025070901). Following a 1-week acclimatization period, mice were randomly divided into five experimental groups (n = 10 per group): Control group: Received normal drinking water and equivalent volume of vehicle by daily oral gavage. DSS group: Administered 3% DSS in drinking water ad libitum for 7 days with vehicle gavage. DSS + HYP 10 mg/kg group: Treated with 3% DSS plus low-dose HYP (10 mg/kg/day) by oral gavage. DSS + HYP 30 mg/kg group: Treated with 3% DSS plus high-dose HYP (30 mg/kg/day) by oral gavage. DSS + 5-aminosalicylic acid (5-ASA) group: Received 3% DSS plus 5-ASA (100 mg/kg/day) by oral gavage. The dosages of HYP used in this study were determined based on previous research (Cheng et al., 2021).

In a separate experiment to evaluate the microbiota-dependent therapeutic effects of HYP, mice were randomly assigned to three experimental groups (n = 5 per group) with the following treatments: ABX group: Received daily oral gavage of a broad-spectrum antibiotic cocktail (1 mg/mL ampicillin, 1 mg/mL metronidazole, 0.5 mg/mL vancomycin, 1 mg/mL neomycin) for 5 consecutive days to deplete gut microbiota, followed by regular drinking water for 7 days. ABX + DSS group: Underwent identical antibiotic pretreatment as the ABX group, followed by 3% DSS administration in drinking water for 7 days to induce colitis under microbiota-depleted conditions. ABX + DSS + HYP group: Received the same antibiotic and DSS regimen as the ABX + DSS group, with concurrent daily oral administration of HYP (30 mg/kg) for 7 days during the DSS exposure period.

### Hematoxylin-eosin staining

Distal colon specimens were fixed in 4% paraformaldehyde for 24 h at 4°C, dehydrated through a graded ethanol series, cleared in xylene, and embedded in paraffin.

Serial 4-μm sections were cut, deparaffinized in xylene, and rehydrated in descending ethanol concentrations. Sections were stained with freshly filtered hematoxylin staining solution (3–5 min) to visualize nuclei, counterstained with eosin staining to highlight cytoplasmic structures, dehydrated through graded ethanol and xylene, and coverslipped with neutral balsam. Histopathological evaluation was performed according to the scoring system described previously (Chen et al., 2025).

### Quantitative real-time PCR analysis

Total RNA was extracted from colon tissues using the FastPure RNA Kit III. Following genomic DNA removal, cDNA was synthesized from 1 μg RNA using the PrimeScript™ RT Reagent Kit (Takara Bio). qRT-PCR assays were performed in triplicate using SYBR Premix Ex Taq II on a QuantStudio 7 Flex system. The thermal cycling conditions were as previously described (Chen et al., 2019). Relative gene expression was calculated via the 2^-ΔΔCT^ method normalized to *Gapdh*. Primer sequences are listed in **Table 1**.

**Table 1:** List of primers of the genes.

Gene	Forward primer	Reverse primer
Gapdh	TCTCTGCTCCTCCCTGTTCT	ATCCGTTCACACCGACCTTC
Tnf-α	ATGGGCTCCCTCTCATCAGT	TGCTTGGTGGTTTGCTACGA
Il-6	CTTCTTGGGACTGATGCTGGT	CTCTGTGAAGTCTCCTCTCCG
Il-1β	GTGTCTTTCCCGTGGACCTT	AATGGGAACGTCACACACCA

### Immunofluorescence and immunohistochemistry

Paraffin-embedded tissue sections were deparaffinized and rehydrated. For immunofluorescence, sections were blocked with BSA and incubated overnight at 4°C with primary antibodies, followed by appropriate secondary antibodies and DAPI counterstaining. For immunohistochemistry, antigen retrieval was performed using EDTA buffer, followed by incubation with primary and secondary antibodies. Signal was developed using DAB substrate, and sections were counterstained with hematoxylin. Images were captured using a fluorescence or bright-field microscope, respectively.

### Enzyme-linked immunosorbent assay

Levels of pro-inflammatory cytokines (IL-6, IL-1β, and TNF-α) in serum were quantified with specific ELISA kits (Cusabio, Wuhan, China). The assay was performed following the manufacturer’s protocols. The absorbance of each sample was measured with a microplate reader, and the corresponding cytokine concentrations were determined by interpolation from a standard curve.

### Open field test for anxiety-like behavior assessment

The open field test was conducted to evaluate anxiety-related behaviors in mice using an established protocol with minor modifications. The testing apparatus consisted of a black polyvinyl chloride square arena (50 × 50 × 40 cm) with white flooring. The testing room was maintained under dim illumination with minimal environmental noise. Each mouse was gently placed in a corner of the arena and allowed to freely explore for 5 minutes (300 seconds). Between trials, the arena was thoroughly cleaned with 75% ethanol to eliminate odor cues. Total distance traveled (mm), average movement velocity (mm/s), time spent in central zone (% of total trial duration) and time spent in peripheral zone (% of total trial duration) were calculated.

### Elevated plus maze test for anxiety assessment

The elevated plus maze test was performed to evaluate anxiety-like behaviors following established protocols with modifications. The apparatus consisted of four perpendicular arms (30 cm long × 5 cm wide) arranged in a “+” configuration elevated 50 cm above the floor. Two opposing arms were enclosed by 15-cm high opaque walls (closed arms), while the other two arms had no walls (open arms). All arms extended from a central square platform (5 × 5 cm). Prior to testing, mice were acclimated to the testing room for 30 minutes under dim light. Each mouse was gently placed in the central platform facing an open arm and allowed to freely explore the maze for 200 seconds. Behavioral sessions were recorded using a video tracking system. Total distance traveled (mm) and average movement velocity (mm/s) was calculated.

### Western blot analysis

Colon tissue proteins were extracted using RIPA lysis buffer supplemented with protease and phosphatase inhibitors (all from Beyotime). Protein concentrations were determined with a BCA assay kit (Proteintech). Equal amounts of protein were separated by 10% SDS-PAGE and transferred to PVDF membranes. After blocking with 5% skim milk, membranes were incubated overnight at 4°C with primary antibodies, followed by incubation with HRP-conjugated secondary antibodies. Protein bands were visualized using an enhanced chemiluminescence detection system (Tanon).

### Metagenomic sequencing and analysis

#### DNA extraction, library preparation, and sequencing

Total microbial genomic DNA was extracted from fecal samples using the OMEGA Mag-Bind Soil DNA Kit (Omega Bio-Tek, USA). DNA quality and quantity were assessed using a Qubit™ 4 Fluorometer (Invitrogen, USA) and agarose gel electrophoresis. Sequencing libraries were prepared with the Illumina TruSeq Nano DNA LT Library Preparation Kit and sequenced on an Illumina NovaSeq platform (Illumina, USA) using a PE150 strategy at Metabo-Profile Biotechnology Co., Ltd. (Shanghai, China).

#### Data preprocessing, assembly, gene prediction, and annotation

Raw sequencing reads were processed to remove adapters using Cutadapt (v1.2.1) and low-quality reads were filtered using FASTP. Host-derived reads were identified and removed by aligning to the host genome with BMTagger. Quality-filtered reads from each sample were assembled *de novo* using Megahit (v1.1.2) with meta-large parameters. Contigs longer than 300 bp were clustered at 95% identity and 90% coverage using MMseqs2. Genes were predicted from the assembled contigs using Prodigel. The predicted coding sequences (CDSs) were clustered at 90% protein sequence identity and 90% coverage using MMseqs2. Gene abundances were estimated by mapping high-quality reads to the predicted gene catalog using Salmon and normalized by the CPM method. For taxonomic annotation, the lowest common ancestor taxonomy of the non-redundant genes was ascertained using MMseqs2 in “taxonomy” mode, with parameters set to “-lca-mode 3 -s 2”, by aligning them against a customized NCBI database (comprising protein sequences of prokaryotic and eukaryotic microorganism from NCBI-nr, and virus from RVDB).

Functional annotation of the non-redundant genes was performed by homology search against the KEGG, EggNOG, and CAZy databases using MMseqs2. Specifically: EggNOG and GO annotations were obtained using EggNOG-mapper (v2). KEGG Orthology (KO) annotations and pathway analysis were performed using KOBAS.

### Metabolomic analysis

Fecal metabolomic profiling was performed using the Q300 Kit (Metabo-Profile, Shanghai, China). Briefly, fecal samples were homogenized with zirconium oxide beads in a mixture of deionized water and methanol containing internal standards. After centrifugation, the supernatant was collected and derivatized at 30 °C for 60 min using a Biomek 4000 workstation (Beckman Coulter, USA). The derivatized samples were reconstituted in 50% methanol and subjected to LC-MS analysis.

Analysis was conducted on an ACQUITY UPLC-Xevo TQ-S system (Waters Corp., USA). Separation was achieved on a BEH C18 column (2.1 × 100 mm, 1.7 μm) maintained at 40°C, with a mobile phase consisting of (A) 0.1% formic acid in water and (B) acetonitrile:isopropanol (70:30). The gradient elution program was as follows: 0–1 min (5% B), 1–11 min (5–78% B), 11–13.5 min (78–95% B), 13.5–14 min (95–100% B), 14–16 min (100% B), 16–16.1 min (100-5% B), 16.1–18 min (5% B), flow rate: 0.40 mL min-1, and injection vol.: 5.0 µL. Mass spectrometry was performed in both ESI positive and negative modes with capillary voltages of 1.5 kV and 2.0 kV, respectively. The source and desolvation temperatures were set at 150°C and 550°C, respectively.

Raw data were processed using the iMAP platform (v1.0, Metabo-Profile). Multivariate analyses, including principal component analysis (PCA) and orthogonal partial least squares-discriminant analysis (OPLS-DA), were performed. Differentially expressed metabolites (DEMs) were identified based on a variable importance in projection (VIP) score of ≥ 1 from the OPLS-DA model and a p-value < 0.05 from univariate analysis.

### Network pharmacology analysis

#### Target identification and screening

Potential targets of Hyperoside were retrieved from the databases: ChEMBL, CTD (https://ctdbase.org/), TTD (http://db.idrblab.net/ttd/), TargetNet (http://targetnet.scbdd.com/home/index/), Swiss-Drugs (https://www.swisstargetprediction.ch/), Drugbank (https://go.drugbank.com/), and PharmMapper (https://www.lilab-ecust.cn/pharmmapper/), with the species limited to “Homo sapiens.” Ulcerative colitis (UC)-related targets were collected from DisGeNet (http://www.disgenet.org/), GeneCards (https://go.drugbank.com/), OMIM (https://omim.org/), DrugBank (https://go.drugbank.com/), CTD (https://ctdbase.org/), and TTD (http://db.idrblab.net/ttd/) databases under the same species restriction. All target gene names were standardized using the UniProt database. The overlapping targets between Hyperoside and UC were identified as potential therapeutic targets and visualized using a Venn diagram.

#### Protein-protein interaction network construction

The common targets were imported into the STRING database (https://string-db.org/) to construct a PPI network, with the species set to “Homo sapiens” and a high confidence (interaction score > 0.7) threshold. Disconnected nodes were hidden in the network. The resulting network was then imported into Cytoscape software. The cytoHubba plugin was used to analyze the network, and the top 20 core targets ranked by composite score were selected as core targets for hyperoside treatment of UC. Visualization of these core target genes was performed using the HiPlot website (https://hiplot-academic.com/basic).

### Enrichment analysis

The common targets were submitted to the Metascape platform for Kyoto Encyclopedia of Genes and Genomes (KEGG) pathway enrichment analysis. The analysis parameters were set as follows: H. sapiens as the species, a p-value cutoff of 0.01, a minimum overlap of 3, and a minimum enrichment of 1.5. The top 20 significantly enriched pathways, ranked by p-value, were selected for visualization. Visualize the KEGG results using the online data analysis and visualization platform Bioinformatics (http://www.bioinformatics.com.cn). Map the top 10 pathways by P-value to the potential functional targets of HYP treatment for UC in a cytoscape-style pathway mapping.

### Molecular docking

The three-dimensional crystal structures of the core target proteins were obtained from the RCSB-PDB database (https://www.rcsb.org/). All protein macromolecular structures were processed using PyMOL v2.5 (open-source version) to remove original ligands, water molecules, and unnecessary ions, retaining only the polypeptide chains necessary for docking. Subsequent preparation, including the addition of hydrogen atoms and assignment of charges, was performed using AutoDockTools to generate the final protein structures in PDBQT format.

The 3D chemical structure of Hyperoside was retrieved from the PubChem database (https://pubchem.ncbi.nlm.nih.gov/), and its SDF format file was downloaded (**Table 2**). Open Babel (v3.1.1) was used for file format conversion and energy minimization. The prepared ligand structure was then processed with AutoDockTools to define rotatable bonds and output in PDBQT format.

**Table 2:** Hyperoside information.

Name	Molecular formula	2D Structure	3D conformer
Hyperoside	C_21_H_20_O_12_	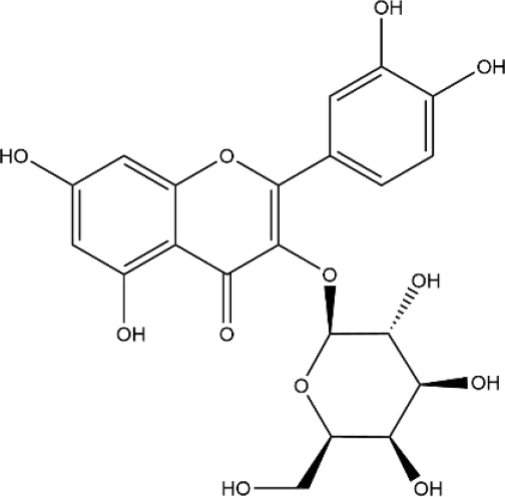	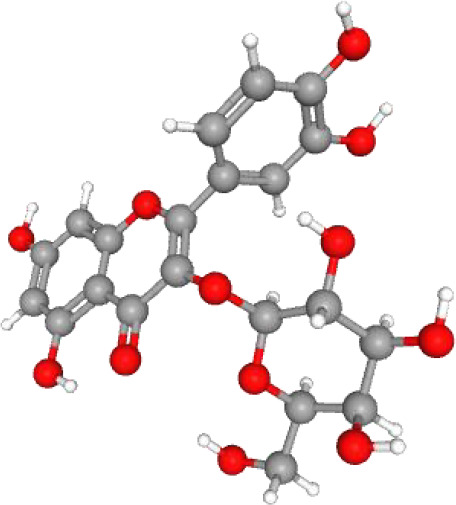

### Docking and interaction analysis

Molecular docking was performed using AutoDock Vina (v1.2.5). The docking grid was configured to encompass the entire known active site or potential binding pocket of each protein. For each target, nine docking poses were generated, and the conformation with the most favorable (lowest) binding energy was selected as the optimal binding mode.

The optimal docking complexes were visualized using PyMOL to analyze the binding conformation and intermolecular interactions, such as hydrogen bonds and hydrophobic contacts. Furthermore, the PoseView server (https://proteins.plus/) was employed to generate a two-dimensional interaction diagram of Hyperoside bound to the protein, enabling analysis of the specific interaction types between key residues and the ligand.

### Statistical analysis

All quantitative data are expressed as mean ± standard deviation (SD). Statistical analyses were performed using GraphPad Prism 8.0. Differences between two groups were assessed using an unpaired, two-tailed Student’s t-test. For comparisons among more than two groups, one-way analysis of variance (ANOVA) was employed. A p-value of less than 0.05 was considered statistically significant (*p < 0.05, **p < 0.01, ***p < 0.001, ****p < 0.0001).

## Results

### HYP ameliorates DSS-induced UC in mice

**Figure 1:**
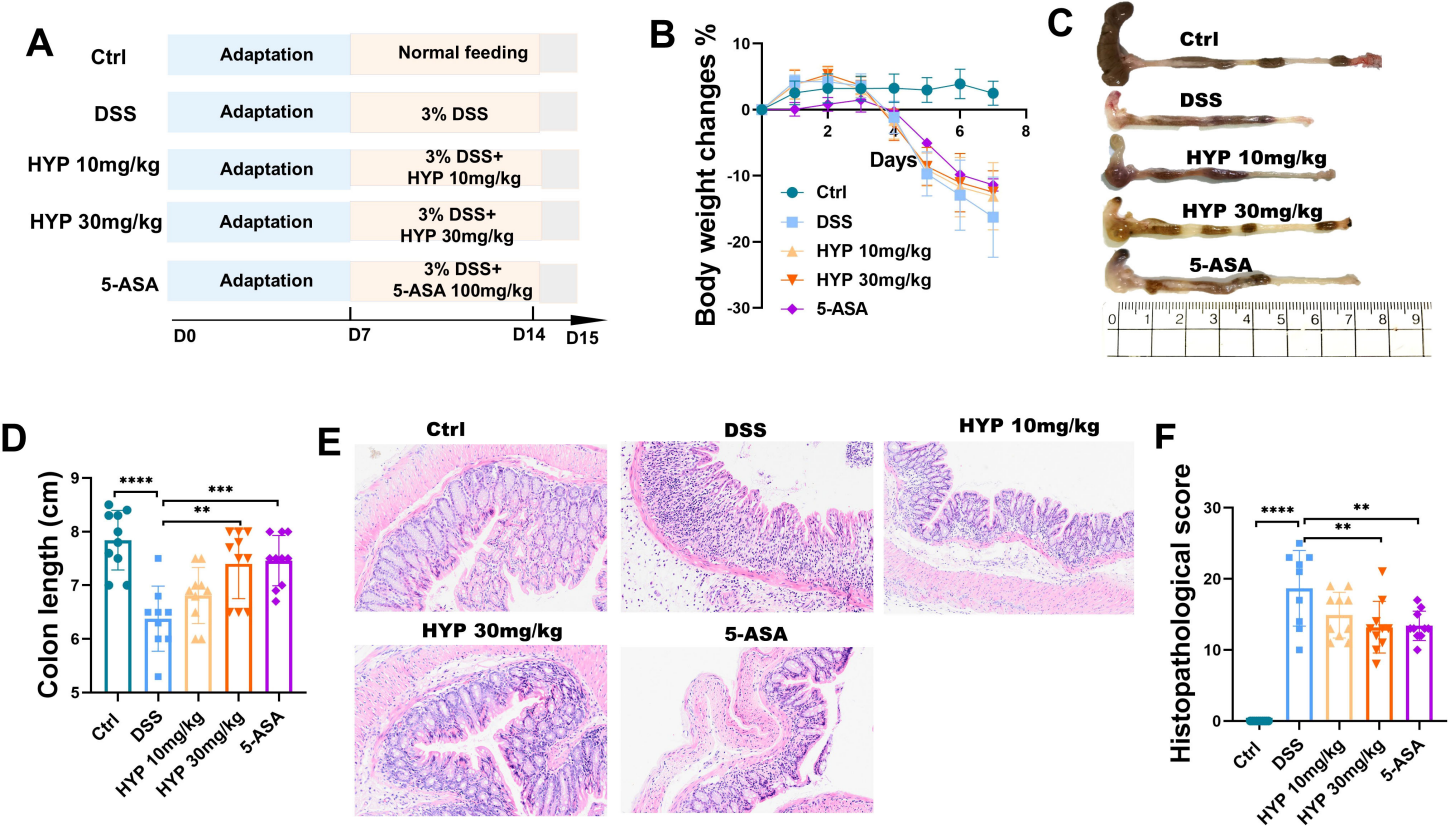
HYP Ameliorates DSS-Induced UC in Mice. **(A)** Schematic diagram of the experimental design. **(B)** Changes in body weight (%) during the experimental period. **(C)** Representative images of colons from each group. **(D)** Quantitative analysis of colon length. **(E)** Representative H&E-stained sections of distal colon tissues. **(F)** Histopathological scores of colons tissues. Data are expressed as means ± SD (n=10). Statistical significance was determined by one-way ANOVA followed by Tukey’s *post hoc* test for multiple comparisons. **P < 0.01 and ****P < 0.0001.

To evaluate the therapeutic effects of HYP on UC, mice first underwent a 7-day acclimatization period. Subsequently, colitis was induced by providing them with 3% DSS in drinking water for 7 days. During the DSS administration period, mice were concurrently treated with either HYP or 5-ASA. The experimental timeline is illustrated in **Figure 1A**. Mice in the DSS group exhibited significant body weight loss compared to the Ctrl group. In contrast, both HYP and 5-ASA treatments markedly attenuated this DSS-induced weight reduction (**Figure 1B**). Furthermore, colon lengths were significantly shorter in the DSS group than in the control group, whereas treatment with a high dose of HYP (30 mg/kg) or 5-ASA substantially reversed this shortening (**Figures 1C, D**). Histological analysis revealed severe mucosal damage in the DSS group, characterized by crypt structure destruction, loss of goblet cells, and substantial inflammatory cell infiltration. These pathological changes were notably alleviated by HYP and 5-ASA treatment, accompanied by significantly lower histopathological scores in the intervention groups (**Figures 1E, F**). Collectively, these findings demonstrate that HYP administration ameliorates DSS-induced UC in mice.

### HYP alleviates anxiety-like behaviors in DSS-induced colitis mice

**Figure 2:**
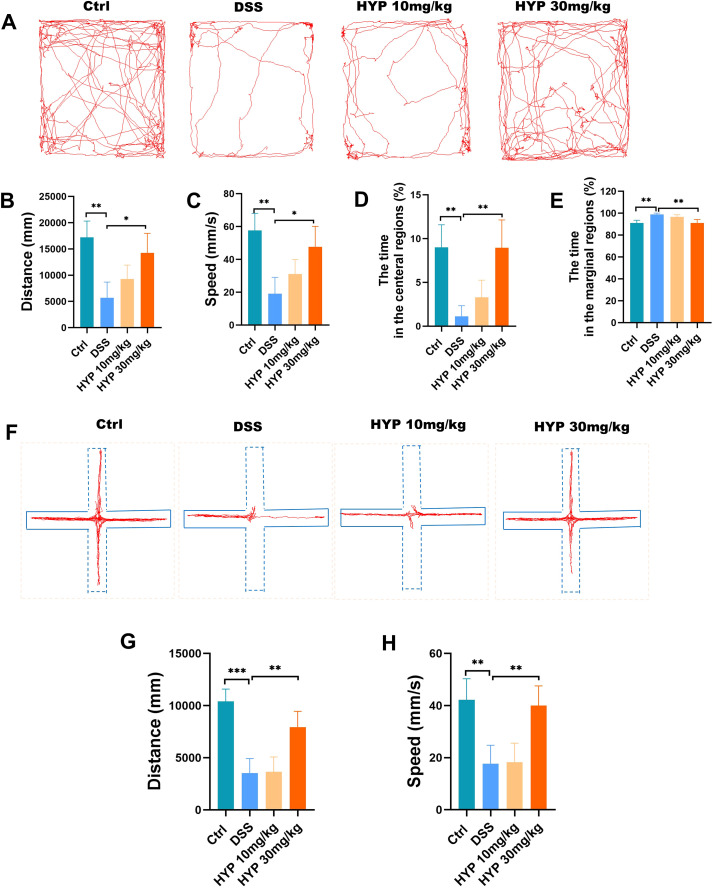
HYP alleviates anxiety-like behaviors in DSS-induced colitis mice. **(A)** Schematic of the open field test (OFT). **(B)** Total moving distance, **(C)** average velocity, **(D)** time spent in the central zone, and **(E)** the time spent in the marginal regions in the OFT. **(F)** Schematic of the elevated plus maze (EPM) test. **(G)** Total moving distance, and **(H)** average velocity in the EPM. Data are expressed as means ± SD (n=4). Statistical significance was determined by one-way ANOVA followed by Tukey’s *post hoc* test for multiple comparisons. *P < 0.05, **P < 0.01, and ***P < 0.001.

To investigate the impact of HYP on UC-associated anxiety and depressive-like behaviors, we performed a series of behavioral tests. In the OFT (**Figure 2A**), DSS-treated mice exhibited a significant reduction in total moving distance (**Figure 2B**), average velocity (**Figure 2C**), and time spent in the central zone (**Figure 2D**) compared to the Ctrl group. These behavioral deficits were markedly reversed by high-dose HYP treatment. Consistent with these findings, DSS administration increased the time spent in the marginal regions of the arena, a trend that was also counteracted by HYP intervention (**Figure 2E**). The EPM test yielded complementary results (**Figure 2F**). Mice in the DSS group showed a significant decrease in both the total distance traveled (**Figure 2G**) and the average movement velocity (**Figure 2H**) compared to controls. High-dose HYP treatment notably restored these activity parameters. Collectively, these behavioral findings demonstrate that HYP treatment effectively attenuates anxiety-like behaviors in a murine model of DSS-induced colitis.

### HYP ameliorates intestinal inflammation and restores barrier integrity in DSS-induced colitis mice

**Figure 3:**
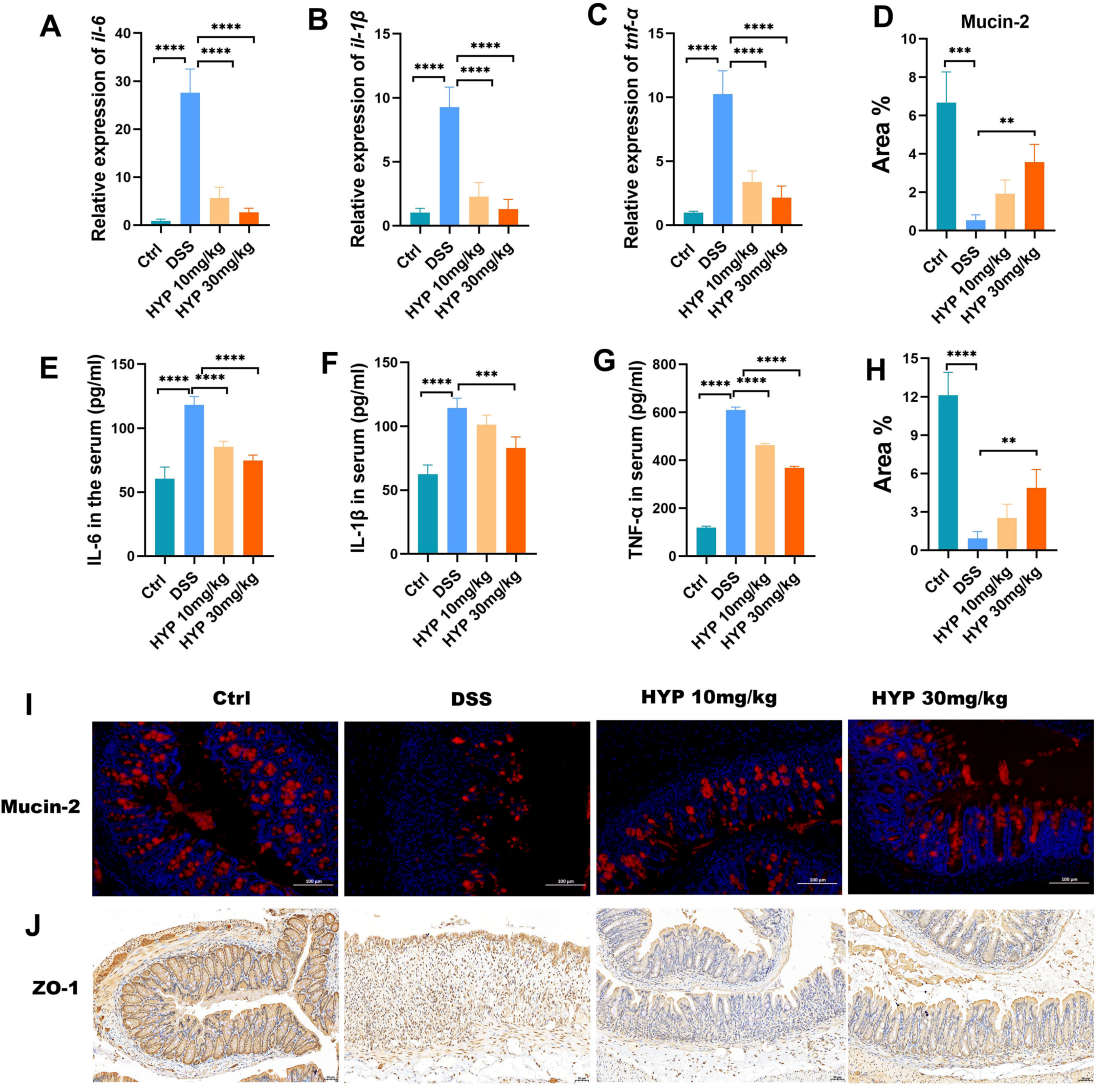
HYP ameliorates intestinal inflammation and restores barrier integrity in DSS-induced colitis mice. mRNA expression levels of **(A)** IL-6, **(B)** IL-1β, and **(C)** TNF-α in colon tissues. Levels of **(E)** IL-6, **(F)** IL-1β, and **(G)** TNF-α in serum. Quantitative analysis of the positive area percentage for **(D)** Mucin-2 and **(H)** ZO-1. Representative images of **(I)** Mucin-2 immunofluorescence and **(J)** ZO-1 immunohistochemistry staining in colon tissues. Data are expressed as means ± SD (n=4-6). Statistical significance was determined by one-way ANOVA followed by Tukey’s *post hoc* test for multiple comparisons. **P < 0.01, ***P < 0.001, and ****P < 0.0001.

To assess the impact of HYP on systemic inflammation, we measured key inflammatory cytokines. DSS administration significantly elevated the levels of pro-inflammatory cytokines, including TNF-α, IL-6, and IL-1β in mouse colon tissues and serum compared with the Ctrl group. Treatment with HYP, however, effectively suppressed these DSS-induced increases (**Figures 3A–C, E-G**). We further investigated whether HYP could protect the intestinal barrier. Immunofluorescence analysis revealed that DSS challenge markedly reduced the protein expression of Mucin-2, a critical component of the mucosal layer, compared to the control group. In contrast, HYP intervention significantly restored Mucin-2 expression (**Figures 3D, I**). Similarly, the expression of the tight junction protein ZO-1 was profoundly decreased by DSS but was notably upregulated after HYP treatment (**Figures 3H, J**). Taken together, these findings demonstrate that HYP ameliorates intestinal inflammation and promotes restoration of barrier integrity in mice with DSS-induced colitis.

### HYP alleviated neuroinflammation in DSS-treated mice

**Figure 4:**
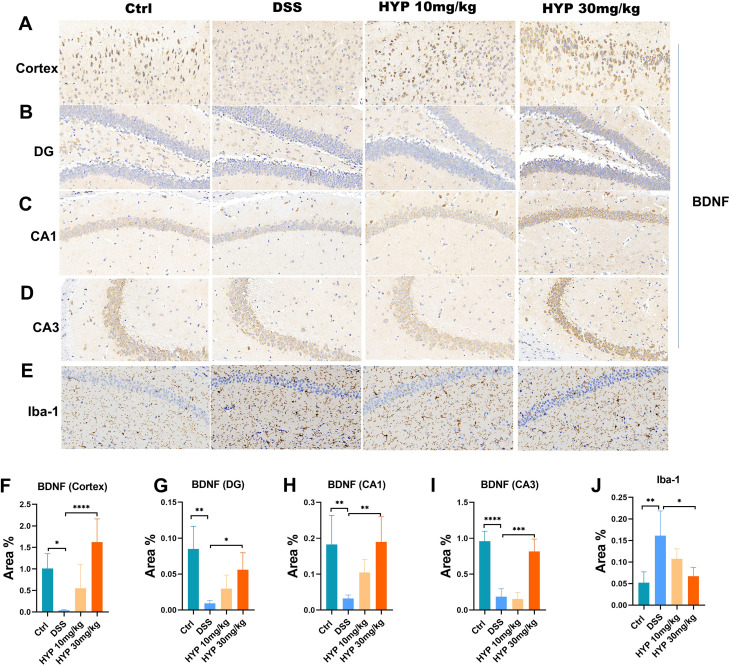
HYP alleviated neuroinflammation in DSS-treated mice. Immunohistochemistry staining of BDNF in the **(A)** cortex, **(B)** DG, **(C)** CA1 and **(D)** CA3 hippocampal regions. **(E)** Representative Iba-1 immunohistochemistry staining. Quantitative analysis of BDNF-positive area percentage in the **(F)** cortex, **(G)** DG, **(H)** CA1 and **(I)** CA3 regions, and **(J)** Iba-1**-**positive area percentage. Data are expressed as means ± SD (n=3-4). Statistical significance was determined by one-way ANOVA followed by Tukey’s *post hoc* test for multiple comparisons. *P < 0.05, **P < 0.01, ***P < 0.001, and ****P < 0.0001.

To determine whether HYP modulates the brain response to peripheral inflammation, we first examined brain histopathology. Hematoxylin and eosin (H&E) staining revealed no overt morphological changes in the hippocampal region across all groups (**Supplementary Figure 1A**). Given the established roles of neurotrophic deficits and glial cell activation in neuroinflammation and anxiety-like behavior, we next assessed the expression of key molecular markers by immunohistochemistry. We analyzed brain-derived neurotrophic factor (BDNF) and the microglial/astrocyte activation markers Iba1 and GFAP, respectively. As shown in **Figure 4**, DSS administration significantly downregulated BDNF protein levels in multiple brain regions, including the cortex (**Figure 4A**), dentate gyrus (DG) (**Figure 4B**), CA1 (**Figure 4C**) and CA3 (**Figure 4D**) hippocampal areas, compared to the Ctrl group. HYP treatment effectively reversed this DSS-induced reduction in BDNF (**Figures 4F-I**). Conversely, DSS challenge markedly upregulated the expression of Iba1, a marker of microglial activation. This increase was significantly suppressed by HYP intervention (**Figures 4E, J**). In contrast, the expression of GFAP, an astrocyte marker, showed no significant alterations among the groups (**Supplementary Figures 1B, C**). These results indicate that HYP mitigates DSS-induced neuroinflammation, likely through the restoration of BDNF expression and suppression of microglial activation.

### HYP reshapes the gut microbiota compositions in DSS-induced UC mice

**Figure 5:**
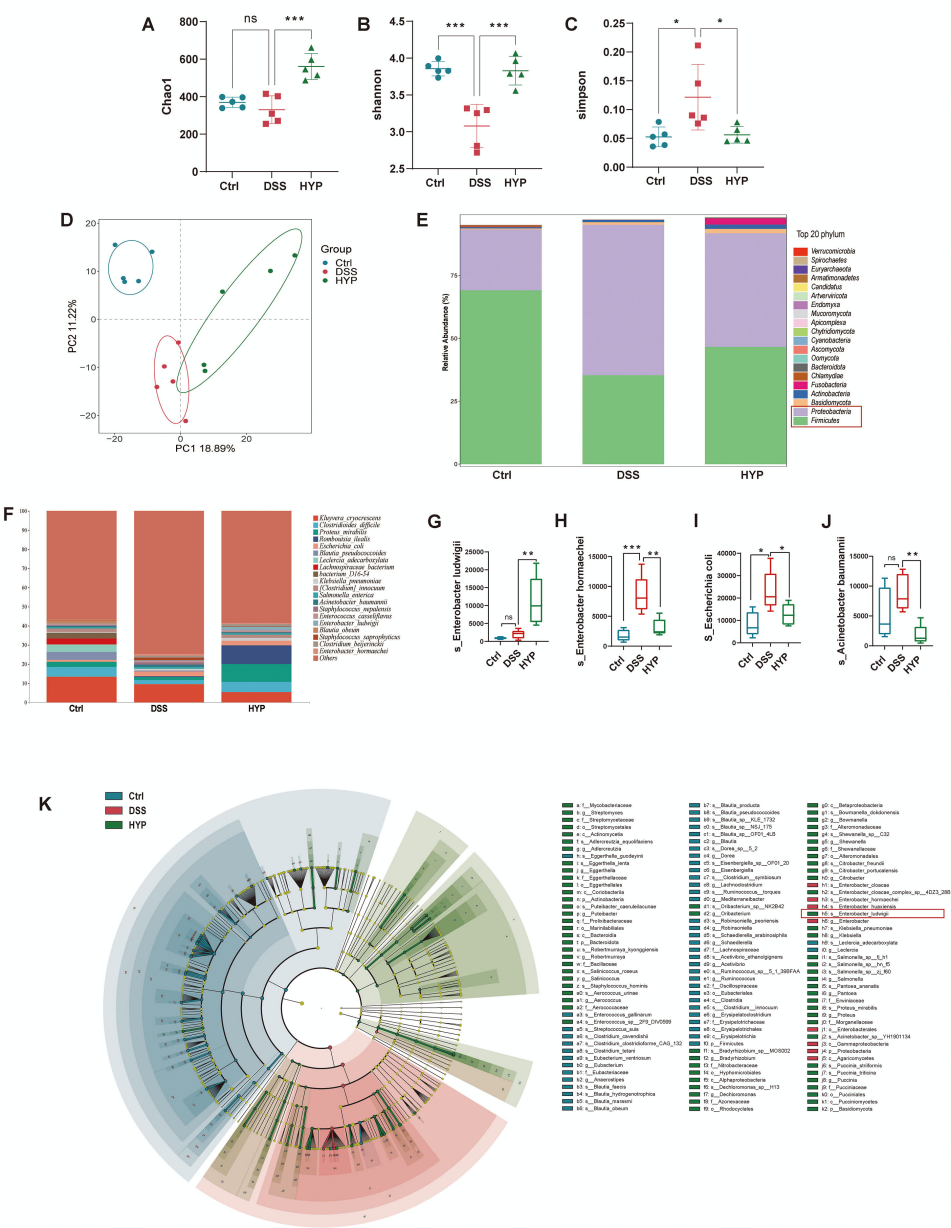
HYP reshapes the gut microbiota compositions in DSS-induced UC mice. Alpha-diversity indices including **(A)** Chao1, **(B)** Shannon, and **(C)** Simpson. **(D)** PCA score plot. Relative abundance of gut microbiota at the **(E)** phylum level and **(F)** species level. Relative abundance of **(G)***Enterobacter ludwigii*, **(H)***Enterobacter hormaechei*, **(I)***Escherichia coli*, and **(J)***Acinetobacter baumannii*. **(K)** Histogram of LDA scores from LEfSe analysis showing differentially abundant taxa among groups. Data are expressed as means ± SD (n=5). Statistical significance was determined by one-way ANOVA followed by Tukey’s *post hoc* test for multiple comparisons. *P < 0.05, **P < 0.01, and ***P < 0.001.

To assess the impact of HYP on the gut microbial community, we first analyzed both alpha and beta diversity. Significant differences were observed in alpha diversity indices, including Chao1 (**Figure 5A**), Shannon (**Figure 5B**), and Simpson (**Figure 5C**), among the Control, DSS, and HYP-treated groups. Principal component analysis (PCA) further revealed distinct clustering of microbial communities, indicating differences in overall composition (beta diversity) across groups (**Figure 5D**). To elucidate the specific taxonomic shifts underlying these changes, we profiled the microbiota at the phylum and species levels. At the phylum level, HYP treatment reversed the DSS-induced microbial imbalance, characterized by a lower relative abundance of Proteobacteria and a higher abundance of Firmicutes, similar to the profile observed in the control group (**Figure 5E**). Further analysis at the species level showed that HYP intervention significantly increased the abundance of *Enterobacter ludwigii* (**Figure 5G**) while reducing the levels of *Enterobacter hormaechei* (**Figure 5H**), *Escherichia coli* (**Figure 5I**), and *Acinetobacter baumannii* (**Figure 5J**). LEfSe analysis (LDA score > 3.0) identified *s:Enterobacter ludwigii* as a key biomarker enriched in the HYP-treated group (**Figure 5K**), suggesting its potential involvement in the therapeutic mechanism of HYP against UC. Notably, *Enterobacter ludwigii* has been previously reported to mitigate colitis, restore intestinal barrier integrity, and promote gut health (Li et al., 2022). Collectively, these findings indicate that HYP administration effectively alleviates DSS-induced dysbiosis and restructures the gut microbiota composition.

### HYP alters the intestinal metabolite profile and promotes arginine biosynthesis

**Figure 6:**
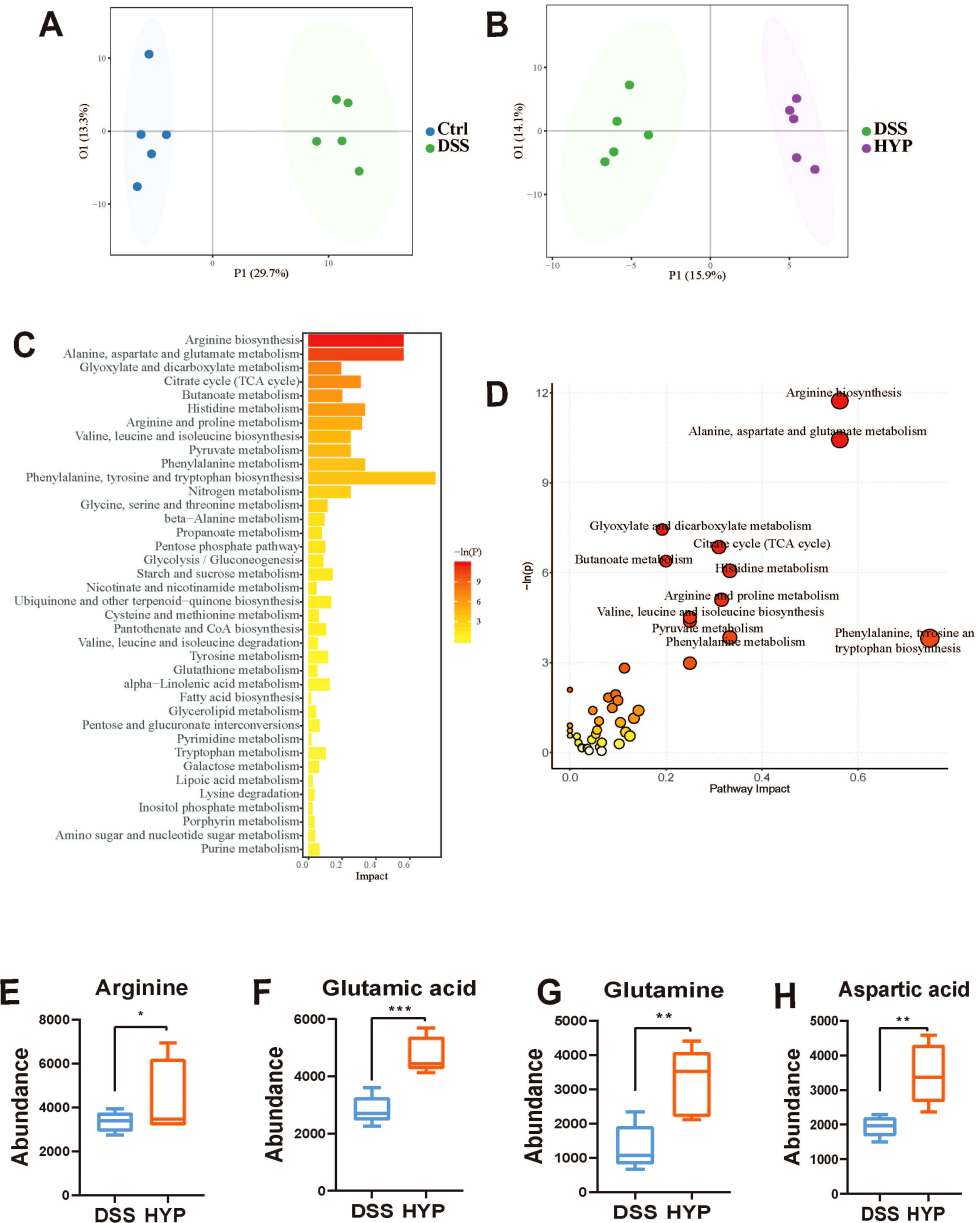
HYP alters the intestinal metabolite profile and promotes arginine biosynthesis. OPLS-DA score plots comparing **(A)** Ctrl vs. DSS and **(B)** DSS vs. HYP groups. **(C, D)** KEGG pathway enrichment analysis of differential metabolites. The abundance of key metabolites of arginine biosynthesis: **(E)** arginine, **(F)** glutamic acid, **(G)** glutamine, and **(H)** aspartic acid. Data are expressed as means ± SD (n=5). Statistical significance for the comparison between DSS and HYP groups was assessed using an unpaired, two-tailed Student’s t-test. *P < 0.05, **P < 0.01, and ***P < 0.001.

To investigate the metabolic alterations underlying the therapeutic effects of HYP, we conducted a comprehensive quantitative metabolomic analysis using the Q300™ platform. Orthogonal partial least squares-discriminant analysis (OPLS-DA) revealed clear metabolic separations between the Ctrl and DSS groups (**Figure 6A**), as well as between the DSS and HYP-treated groups (**Figure 6B**), indicating a significant shift in the global metabolite profile following HYP intervention. Subsequent KEGG pathway enrichment analysis of the differentially abundant metabolites showed that HYP treatment significantly enriched several metabolic pathways. Most notably, the arginine biosynthesis pathway was prominently enriched, along with pathways for alanine, aspartate, and glutamate metabolism, and glyoxylate and dicarboxylate metabolism (**Figures 6C, D**). Consistent with the pathway analysis, HYP intervention significantly increased the relative abundances of key metabolites within the arginine biosynthesis pathway, including arginine (**Figure 6E**), glutamic acid (**Figure 6F**), glutamine (**Figure 6G**), and aspartic acid (**Figure 6H**). Taken together, these findings demonstrate that HYP remodels the host-microbiota metabolome and specifically enhances arginine metabolism, which may contribute to its protective effects against UC.

### Network pharmacology and molecular docking elucidate potential mechanisms of HYP in UC treatment

**Figure 7:**
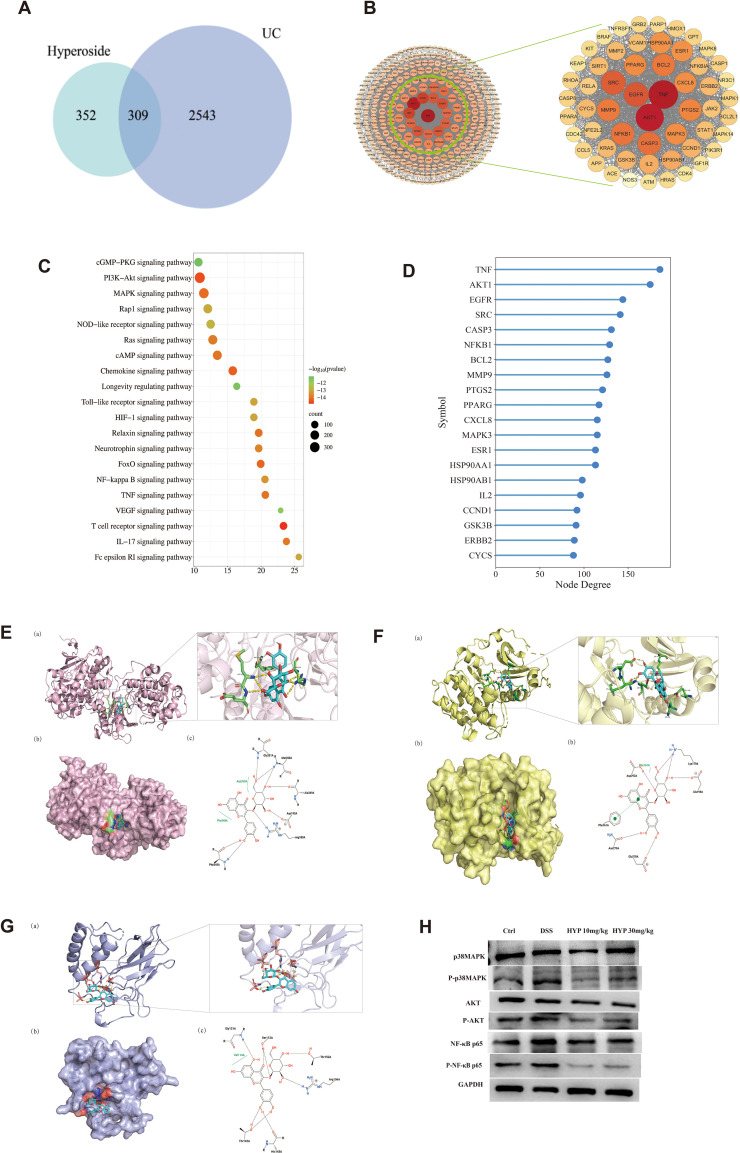
Network pharmacology and molecular docking elucidate potential mechanisms of HYP in UC treatment. **(A)** Venn diagram of HYP and UC target genes. **(B)** PPI network of common targets. **(C)** KEGG pathway analysis. **(D)** The top 20 gene targets ranked by degree value. Molecular docking binding modes and affinities of HYP with **(E)** MAPK3, **(F)** AKT1, and **(G)** NFκB1. **(H)** Protein expression levels of p38MAPK, p-p38MAPK, AKT, p-AKT, NF-κB p65, and p-NF-κB p65 in the colon tissues.

To systematically investigate the underlying mechanisms of HYP against ulcerative colitis (UC), we performed a network pharmacology analysis. A total of 661 HYP-related targets and 2,852 UC-related targets were identified, with 309 overlapping targets considered as potential therapeutic targets of HYP for UC (**Figure 7A**). A protein-protein interaction (PPI) network was constructed using these common targets (**Figure 7B**). Subsequent KEGG pathway enrichment analysis revealed that significantly enriched pathways included the PI3K-Akt, MAPK, and NF-κB signaling pathways, all of which have been previously implicated in UC pathogenesis (**Figure 7C**). The top 20 hub genes were identified through topological analysis based on degree values (**Figure 7D**). From these hub genes, three key candidates (MAPK3, AKT1, and NFKB1) were selected for subsequent molecular docking and experimental validation.

Molecular docking was performed to evaluate the binding affinity between HYP and these key proteins. MAPK3 showed the strongest binding affinity with HYP (−8.745 kcal·mol^-1^) (**Figure 7E**), followed by AKT1 (−8.23 kcal·mol^-1^) (**Figure 7F**) and NFκB1 (−7.31 kcal·mol^-1^) (**Figure 7G**). The information of HYP is shown in **Table 2**. The optimal docking pose for each protein is summarized in **Table 3**, with all nine docking poses provided in **Supplementary Table 1**. To further validate these findings, Western blot analysis was conducted to assess the expression of pathway-related proteins (**Figure 7I**). These integrated results suggest that the therapeutic effects of HYP on UC may be mediated through the modulation of multiple signaling pathways, particularly the MAPK, PI3K-Akt, and NF-κB pathways.

**Table 3:** The optimal docking energy for each protein-hyperoside complex.

Gene names	Protein names	PDB ID	Resolution	Highest affinity (kcal·mol^-1^)	Explore in 3D
AKT1	RAC-alpha serine/threonine-protein kinase	4EKL	2.00 Å	-8.23	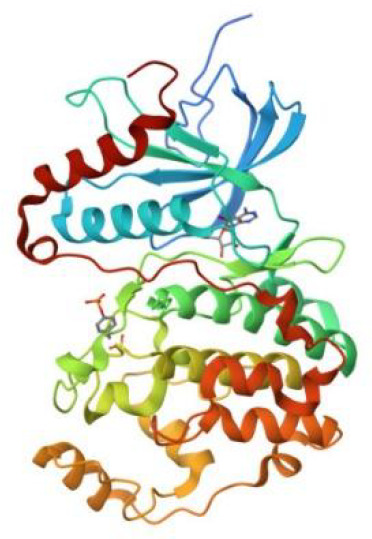
MAPK3	Mitogen-activated protein kinase 3	2ZOQ	2.39 Å	-8.745	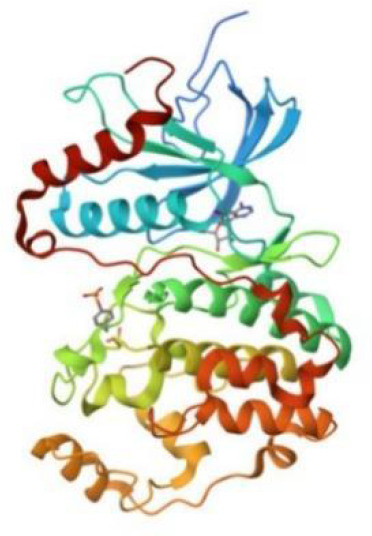
NFκB1	Nuclear factor NF-kappa-B p105 subunit	8TQD	2.02 Å	-7.31	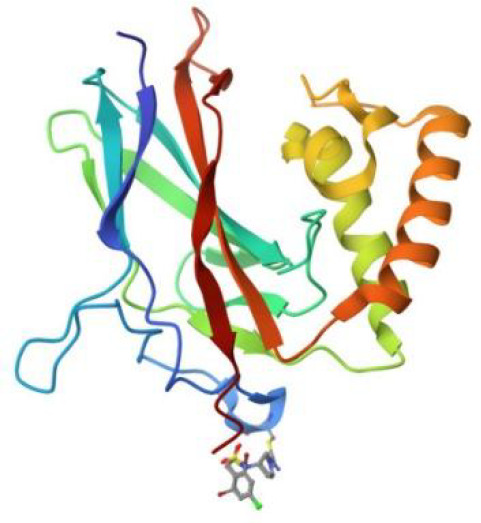

### The therapeutic effects of HYP on colitis and anxiety-like behaviors are microbiota-dependent

**Figure 8:**
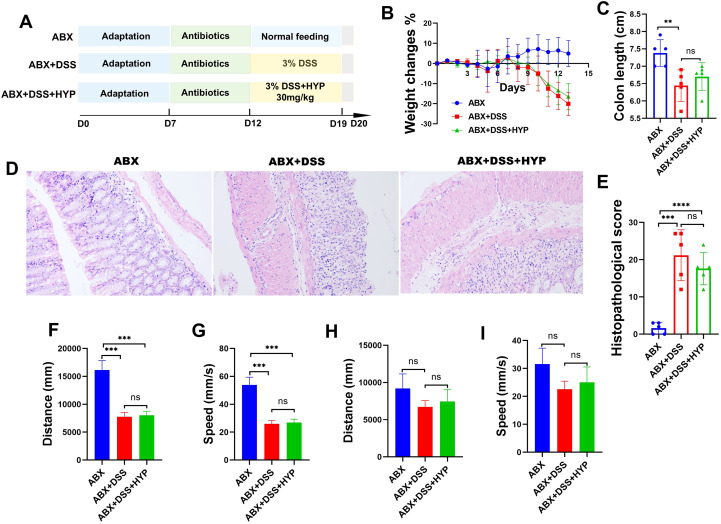
Therapeutic effects of HYP on colitis and anxiety-like behaviors are microbiota-dependent. **(A)** Experimental design of antibiotic intervention. **(B)** Body weight changes (%) during the experimental period. **(C)** Quantitative analysis of colon length. **(D)** Representative H&E-stained sections of distal colon tissues. **(E)** Histopathological scores of colons tissues. **(F)** Moving distance and **(G)** average velocity in the OFT. **(H)** Moving distance and **(I)** average velocity in the EPM test. Data are expressed as means ± SD (n=3-5). Statistical significance was determined by one-way ANOVA followed by Tukey’s *post hoc* test for multiple comparisons. **P < 0.01, ***P < 0.001 and ****P < 0.0001.

To determine whether the intestinal microbiota is required for the therapeutic effects of HYP, we employed an antibiotic depletion strategy (**Figure 8A**). In microbiota-depleted mice, HYP treatment failed to ameliorate DSS-induced body weight loss (**Figure 8B**) or colon shortening (**Figure 8C**). Consistent with this, histopathological analysis showed that HYP no longer mitigated DSS-induced crypt damage and inflammatory infiltration after antibiotic treatment (**Figures 8D, E**). We further assessed whether the behavioral benefits of HYP were also microbiota-dependent. In the OFT, HYP did not improve the reduced moving distance or average velocity in microbiota-depleted mice (**Figures 8F, G**). Similarly, in the EPM test, the restorative effects of HYP on total distance traveled and movement velocity were abolished following antibiotic treatment (**Figures 8H, I**). Collectively, these findings demonstrate that the intestinal microbiota is essential for the efficacy of HYP in alleviating both colitic pathology and associated anxiety-like behaviors.

## Discussion

**Figure 9:**
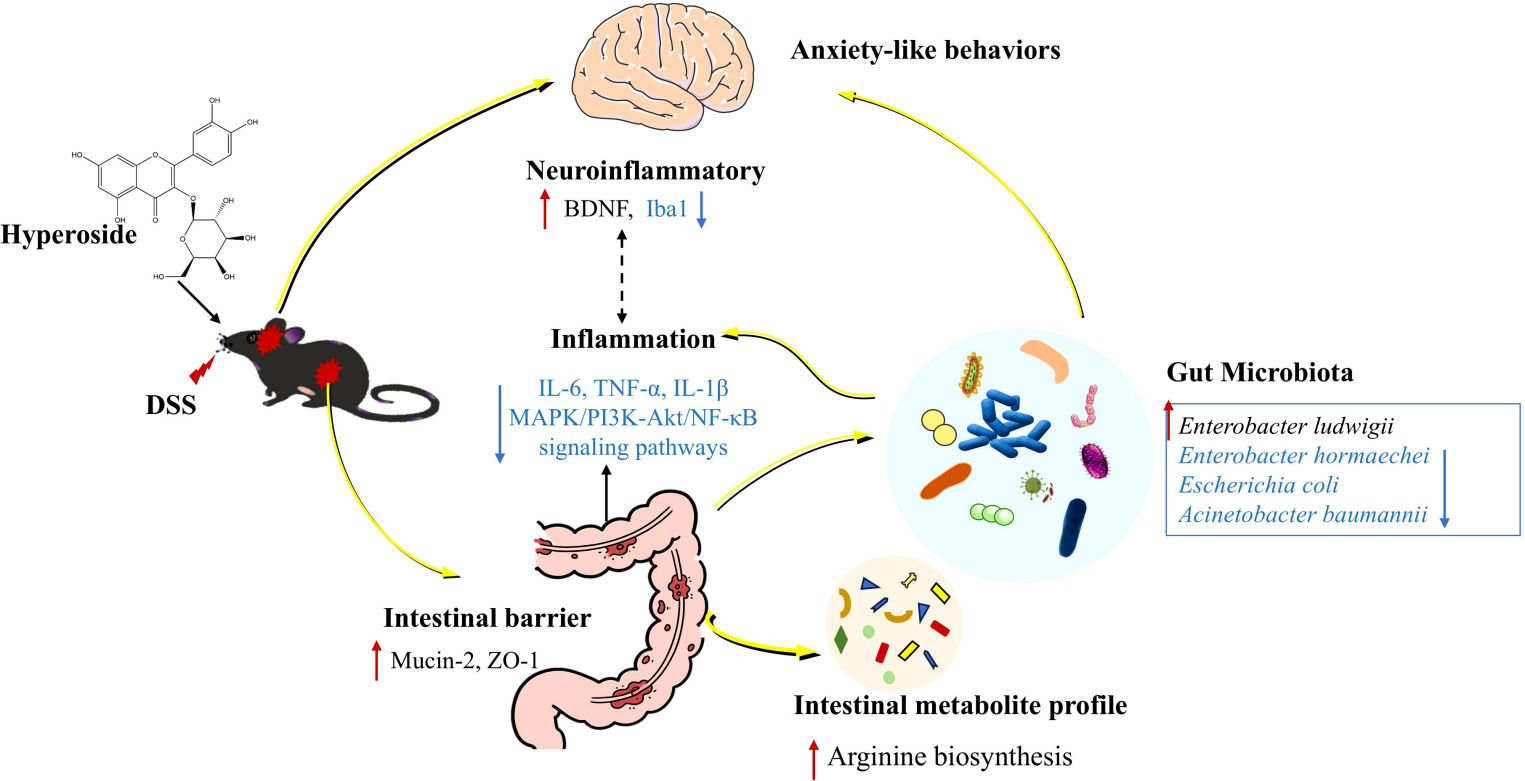
Schematic of the mechanism by which HYP ameliorates DSS-induced UC and associated anxiety-like behaviors. HYP alleviates UC and comorbid anxiety through gut microbiota-dependent mechanisms. It remodels the gut microbial community, thereby enhancing host arginine metabolism and directly modulating the MAPK/PI3K-Akt/NF-κB signaling pathways. This integrated action suppresses systemic and neuroinflammation, restores intestinal barrier function, and ultimately ameliorates both colonic pathology and anxiety-like behaviors.

This study comprehensively demonstrates that the natural flavonoid HYP effectively ameliorates DSS-induced ulcerative colitis and concomitant anxiety-like behaviors in mice, and its therapeutic efficacy is fundamentally dependent on the integrity of the gut microbiota. Our findings delineate a potential mechanistic pathway whereby HYP remodels the gut microbial community, which in turn modulates host arginine metabolism and systemic inflammation, ultimately leading to the restoration of intestinal barrier function and mitigation of neuroinflammation viaxthe MAPK, PI3K-Akt, and NF-κB signaling pathways (**Figure 9**).

The pathogenesis of UC involves a complex interplay of immune dysregulation, epithelial barrier dysfunction, and environmental triggers. Pro-inflammatory cytokines such as TNF-α, IL-1β, and IL-6 are well-established mediators of intestinal inflammation, amplifying tissue injury and sustaining disease activity (Moldoveanu et al., 2015). Concurrently, disruption of the intestinal barrier, a critical defense against luminal pathogens and antigens, is a hallmark of UC pathogenesis (Groschwitz and Hogan, 2009; McGuckin et al., 2009). In line with these concepts, we observed that HYP treatment not only suppressed the production of pro-inflammatory cytokines but also significantly upregulated the expression of key barrier proteins. The restoration of ZO-1, a pivotal tight junction protein (Qian et al., 2025), and Mucin-2, the primary component of the protective mucus layer secreted by goblet cells (Yao et al., 2021), underscores HYP’s dual mechanism of action: mitigating inflammation while actively promoting mucosal healing. These findings corroborate previous reports on the protective effects of HYP on intestinal integrity (Cheng et al., 2021).

Clinical evidence consistently demonstrates that patients with UC show heightened susceptibility to psychiatric comorbidities, particularly anxiety and depression, compared to the general population (Mikocka-Walus et al., 2016; Joo et al., 2024). Mirroring these clinical observations, DSS-induced UC mouse models also exhibit anxiety- and depression-like behaviors (Xie et al., 2022; Liu et al., 2024; Qian et al., 2025). The pathophysiology of these neuropsychiatric symptoms is increasingly linked to neuroinflammation, characterized by aberrant activation of glial cells (Rooney et al., 2020; Al Omran et al., 2022; Novakovic et al., 2023; Lu et al., 2024). As resident immune cells in the central nervous system, microglia play crucial roles in synaptic modulation and neuroimmune balance. Their dysregulation, marked by elevated Iba-1 expression, can promote pro-inflammatory polarization and exacerbate neural dysfunction (Zheng et al., 2021; Lu et al., 2024). Concurrently, BDNF, essential for synaptic plasticity and neuronal survival (Poo, 2001; Wang et al., 2022), demonstrates anti-inflammatory properties through NF-κB suppression (Cai et al., 2014). BDNF deficiency worsens neuroinflammatory and behavioral deficits (Gong et al., 2024), underscoring its central role in mood regulation. In the present study, HYP treatment effectively rebalanced this neuroinflammatory milieu by downregulating Iba-1 expression while upregulating BDNF levels. This coordinated regulation suggests that HYP ameliorates behavioral deficits in DSS-induced UC mice through dual mechanisms of restoring glial homeostasis and enhancing BDNF-mediated neuroprotection, thereby providing a molecular basis for its beneficial effects along the gut-brain axis.

A pivotal finding of this research is the indispensable role of the gut microbiota in mediating the therapeutic effects of HYP. Our analysis revealed that HYP administration significantly reshaped the gut microbial composition, counteracting DSS-induced dysbiosis. Notably, HYP promoted the abundance of *Enterobacter ludwigii*, a species previously identified to have prophylactic and therapeutic effects on DSS-induced colitis by inducing immune tolerance and Treg differentiation (Li et al., 2022). Conversely, HYP reduced the levels of several opportunistic pathogens, including *Enterobacter hormaechei*, *Escherichia coli*, and *Acinetobacter baumannii*. *Escherichia coli* is a major gut microbiome signature strongly associated with UC pathogenesis (Khorsand et al., 2022). *Acinetobacter baumannii* is a nosocomial pathogen known to cause severe infections in immunocompromised hosts (Harding et al., 2018). Most critically, the functional necessity of the gut microbiota was demonstrated through antibiotic depletion experiments. The complete abolition of HYP’s protective effects against both colitis and anxiety-like behaviors in microbiota-deficient mice provides direct evidence that the gut microbiota is not a passive bystander but an essential mediator of HYP’s therapeutic action.

Metabolites are crucial signaling molecules in host-microbe interactions. Our metabolomic analysis revealed that HYP intervention significantly enriched several metabolic pathways, most notably arginine biosynthesis and alanine, aspartate, and glutamate metabolism. The latter has been consistently reported as disrupted in UC (Wang et al., 2022; Wang et al., 2023), aligning with our observations. However, the most pronounced effect of HYP was on the arginine biosynthetic pathway. Arginine is a multifunctional molecule involved in immune regulation, neurotransmission, and tissue repair (Wei et al., 2025). Notably, colonic L-arginine levels are significantly reduced in active UC patients (Coburn et al., 2016), and its supplementation ameliorates experimental colitis (Andrade et al., 2016), underscoring its pathophysiological relevance. The fact that other natural products also exert benefits in UC by enhancing arginine biosynthesis (Wei et al., 2025), corroborates the significance of our finding. Moreover, the reported anxiolytic effects of L-arginine (Lakhan and Vieira, 2010) suggest this pathway may partly mediate the gut-brain axis effects observed. It should be noted that arginine metabolism has complex, context-dependent roles in colitis. While endogenous enhancement appears beneficial, exogenous arginine administration and the rate-limiting enzyme argininosuccinate synthetase 1 (ASS1) overexpression have been reported to exacerbate colitis in some models (Liu et al., 2025), indicating precise regulatory control. In our study, HYP treatment increased the levels of arginine and its precursors (glutamate, glutamine, aspartate), suggesting a coordinated upregulation of this pathway. This provides a plausible mechanism whereby HYP-reshaped microbiota promotes mucosal healing and behavioral improvement. Future research should definitively establish causality, for instance, by testing if arginine (or metabolite) supplementation recapitulates HYP’s benefits and by employing gnotobiotic or fecal microbiota transplantation (FMT) models to pinpoint essential bacterial contributors.

Substantial evidence implicates the dysregulation of key inflammatory signaling pathways, particularly MAPK, PI3K/Akt, and NF-κB, in the pathogenesis of UC (Li et al., 2023; Ma et al., 2024; Qiu et al., 2024; Wang et al., 2024). Among these, Akt serves as a critical upstream regulator that activates NF-κB signaling, a process mediated through phosphorylation of IκB and p65, leading to NF-κB nuclear translocation and subsequent transcription of pro-inflammatory genes (Mostafa and Abdel-Rahman, 2023). Our integrated approach combining network pharmacology and molecular docking provides a systems-level understanding of HYP’s mechanism. The identification of PI3K/Akt, MAPK, and NF-κB pathways as potential targets of HYP aligns precisely with their established roles in driving intestinal inflammation. Molecular docking studies confirmed strong binding affinities between HYP and core signaling components (MAPK3, AKT1, and NFKB1), suggesting that HYP may directly interact with key nodes within these pro-inflammatory cascades. This multi-target engagement capability represents a hallmark of many effective natural products, positioning HYP as a promising modulator of the complex inflammatory network in UC.

This study provides novel insights into the effects of HYP on UC and comorbid anxiety; however, several limitations should be acknowledged. The behavioral and selected molecular analyses were performed with a relatively small sample size. Although such group sizes are typical in exploratory preclinical studies and yielded statistically significant results for primary endpoints, they may reduce the sensitivity to detect more subtle phenotypic differences. Future studies with larger sample sizes will be important to confirm and extend these findings. Furthermore, while our data indicate correlations between HYP administration, microbial alterations, arginine biosynthesis, and behavioral improvement, the precise metabolic fate of HYP in the gut, the functional roles of microbiota-derived metabolites, and the direct causal relationships linking specific microbial changes to arginine metabolic shifts and downstream signaling modulation remain to be fully elucidated. Investigation into these mechanisms will be an important direction for future research.

## Conclusion

In conclusion, our study elucidates that HYP alleviates UC and comorbid anxiety by orchestrating a multi-system response centered on the gut microbiota. HYP remodels the microbial community, which in turn modulates host arginine metabolism and systemic inflammation, ultimately leading to the restoration of intestinal barrier function and mitigation of neuroinflammation via the modulation of PI3K/Akt, MAPK, and NF-κB signaling pathways. These findings position HYP as a promising microbiota-targeting therapeutic candidate for UC and its neuropsychiatric comorbidities.

## Data Availability

The raw data supporting the conclusions of this article will be made available by the authors, without undue reservation.
