# Supplementary material for: Microbiota-driven therapeutic efficacy of Hyperoside in ulcerative colitis and associated anxiety
Source: Front Cell Infect Microbiol. 2026 Jan 29;16:1734356. doi: 10.3389/fcimb.2026.1734356 (PMC12894414; doi:10.3389/fcimb.2026.1734356)
Supplement: Supplementary file 1 [file Table1.docx]

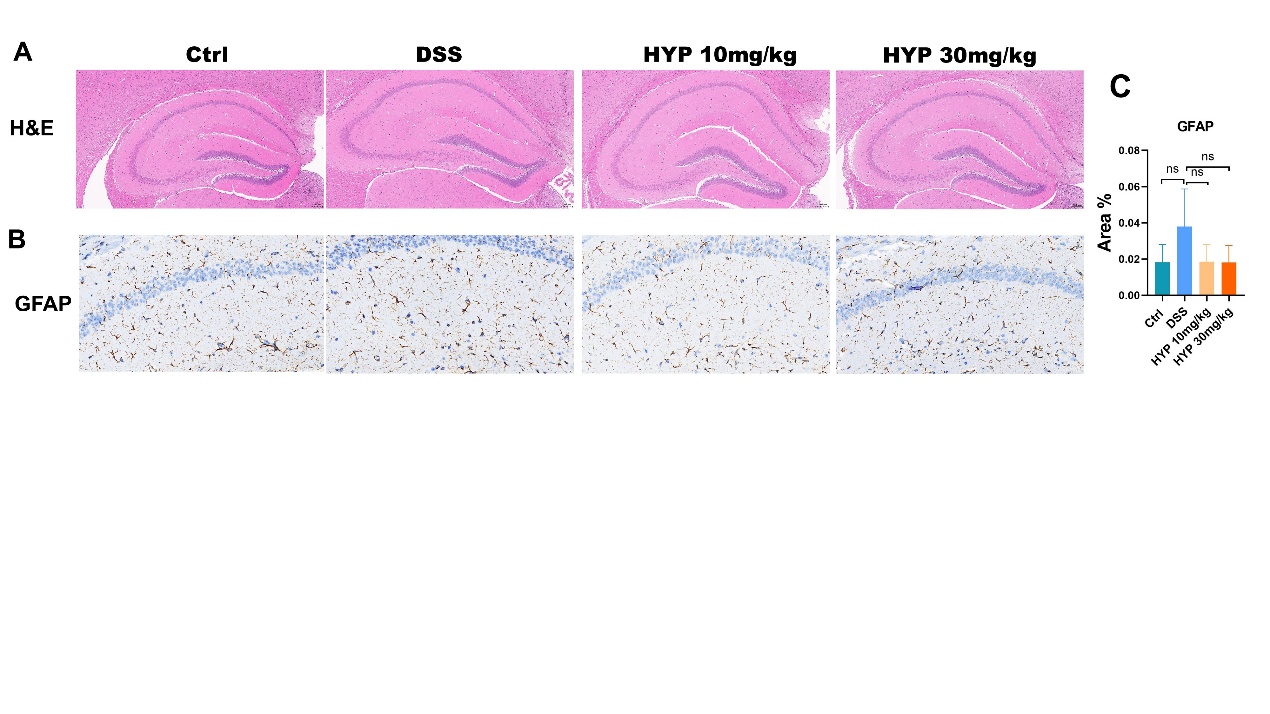


**Supplementary Fig**. 1 **(A)** Representative images of brain tissues with HE staining.

**(B)** Immunohistochemistry staining of GFAP. **(C)**The GFAP-positive area percentage.

Data are expressed as means ± SD (n=4-5).

**Supplementary Table 1 Molecular Docking Simulation Binding Energy**

| PDB ID | Mode | Affinity(kcal·mol⁻¹) |
| --- | --- | --- |
| 4EKL | 1 | -8.23 |
|  | 2 | -7.814 |
|  | 3 | -7.46 |
|  | 4 | -7.284 |
|  | 5 | -7.252 |
|  | 6 | -7.196 |
|  | 7 | -6.586 |
|  | 8 | -6.52 |
|  | 9 | -6.472 |
| 2ZOQ | 1 | -8.745 |
|  | 2 | -8.723 |
|  | 3 | -8.534 |
|  | 4 | -8.478 |
|  | 5 | -8.262 |
|  | 6 | -8.194 |
|  | 7 | -8.148 |
|  | 8 | -8.03 |
|  | 9 | -8.025 |
| 8TQD | 1 | -7.31 |
|  | 2 | -6.933 |
|  | 3 | -6.635 |
|  | 4 | -6.609 |
|  | 5 | -6.594 |
|  | 6 | -6.581 |
|  | 7 | -6.572 |
|  | 8 | -6.553 |
|  | 9 | -6.506 |
